# Genetic Characterization and Zoonotic Potential of *Leptospira interrogans* Identified in Small Non-Flying Mammals from Southeastern Atlantic Forest, Brazil

**DOI:** 10.3390/tropicalmed10030062

**Published:** 2025-02-27

**Authors:** Maria Isabel Nogueira Di Azevedo, Ana Clara dos Reis Soares, Camila Ezepha, Filipe Anibal Carvalho-Costa, Anahi Souto Vieira, Walter Lilenbaum

**Affiliations:** 1 Laboratory of Veterinary Bacteriology, Biomedical Institute, Fluminense Federal University, Niteroi 24020-150, Brazil; anacdrs@id.uff.br (A.C.d.R.S.); camilaezepha@id.uff.br (C.E.); wlilenbaum@id.uff.br (W.L.); 2 Laboratory of Epidemiology and Molecular Systematics, Oswaldo Cruz Institute, Rio de Janeiro 21040-900, Brazil; carvalhocosta70@hotmail.com; 3 School of Veterinary Medicine, Federal University of Mato Grosso do Sul—Paranaíba Campus, Mato Grosso do Sul 79070-900, Brazil; anahi.vieira@ufms.br

**Keywords:** leptospirosis, zoonosis, one health, *Akodon*, *secY*

## Abstract

Leptospirosis is a zoonotic disease of global public health importance caused by bacteria of the genus *Leptospira*. Small non-flying mammals are important reservoirs of the pathogen. The Brazilian Atlantic Forest is a biodiversity hotspot located in a densely populated area and subject to intense degradation. Although documented through serosurveys and the detection of leptospiral DNA in wild small mammals, no study has performed a genetic characterization of the bacteria in the region. The present study aimed to evaluate the genetic diversity of pathogenic leptospires identified in small non-flying mammals in the Southeast Atlantic Forest and to perform intraspecific genetic inferences with other hosts. The studied area included five different conservation units. Molecular diagnosis was performed based on the *lipl32* gene. The SLST typing method was applied based on the *secY* gene. In total, 56% of samples were *lipL32*-PCR-positive and identified as *L. interrogans*, with a high genetic identity among them, distributed in four main haplogroups. The largest haplogroup also included reference sequences from humans, dogs, and urban rats, all belonging to the Icterohaemorrhagiae serogroup. Our results reinforce the role of small mammals as important carriers of *L. interrogans* and highlight the Atlantic Forest as a significant environment for the circulation and dissemination of spirochetes with zoonotic potential.

## 1. Introduction

Leptospirosis is a globally neglected, reemerging zoonotic disease of global public health importance caused by pathogenic spirochetal bacteria of the genus *Leptospira* that circulates among a wide spectrum of hosts, including humans and domestic and wild animals [1]. Leptospires can survive in the environment, particularly in moist conditions, and can be transmitted through different routes: (i) direct contact with the urine of an infected animal; (ii) indirect contact with contaminated water, soil, or feed; and (iii) sexual and vertical transmission in some species (e.g., bovine) [2].

Historically, the etiology of leptospiral infections can be divided into those determined by host-adapted or incidental strains [3]. Adapted strains are observed in the reservoir animal host, resulting from a long process of host–pathogen coevolution, leading to asymptomatic or inapparent disease or mild and subclinical symptoms of a chronic nature [3]. These maintenance hosts are infected asymptomatic animals that shed viable leptospires in their fluids, acting simultaneously as hosts and reservoirs. They play an important role in the epidemiology of leptospirosis, harboring and shedding leptospires in the environment for long periods, becoming important sources of leptospiral infection for non-adapted hosts [4]. Examples of adapted serovars are Bratislava in horses, Pomona in pigs, Hardjo in ruminants, Canicola in dogs, and Icterohaemorrhagiae in rodents [5]. On the other hand, incidental serovars infect hosts other than the maintenance ones, leading to an apparent acute, often severe illness [4]. Importantly, the *L. interrogans* serogroup Icterohaemorrhagiae is the most associated with acute human disease [6], highlighting the incidental nature of infection in people.

The clinical signs of leptospirosis in humans and animals are diverse and can affect multiple organ systems. In humans, the disease can present with a wide spectrum of symptoms, ranging from mild, flu-like symptoms to severe, life-threatening conditions such as Weil’s disease, characterized by jaundice, renal failure, and hemorrhagic manifestations [7]. The bacteria enter through abrasions or cuts in the skin and mucous membranes, including the conjunctiva [8]. Macerated skin, resulting from prolonged exposure to water, is also a suspected route of infection [9].

Small non-flying mammals, such as rodents and marsupials, play a crucial role in the epidemiology of leptospirosis and act as reservoirs of *Leptospira* spp. [8]. These animals shed the bacteria in their urine, contaminating the environment, particularly water sources. This can lead to human infection through direct or indirect contact with contaminated water or soil, representing a constant risk, especially in urban and peri-urban environments [10].

Due to its high biodiversity and species endemism in a small remaining area, the Brazilian Atlantic Forest is among the world’s 36 biodiversity hotspots [11]. It harbors a wide range of mammalian hosts, which provide favorable biotic conditions for the bacterial cycle. In addition, it is located in a tropical region and presents ideal environmental abiotic conditions, such as a hot and humid climate, that favor the survival of leptospires [12]. Several wild and domestic animals live in the region, including 72% of the Brazilian population [13]. Leptospirosis is endemic in this region, with an average of 3810 cases reported annually and with higher numbers in urban areas, including the Rio de Janeiro state, located in the southeastern region of the Atlantic Forest, where the disease remains a significant public health concern, with periodic outbreaks, particularly during the rainy season [14]. Poverty and urbanization, among other environmental and socioeconomic factors, influence morbidity and mortality rates [15].

The few studies that have documented the occurrence of *Leptospira* spp. in small mammals in the Atlantic Forest [16,17,18,19] were based on serosurveys, which present limited value since reservoirs may not be detected by serology [20]. Previous studies conducted by our group in the region have identified the presence of pathogenic leptospiral DNA in wild marsupials and rodents based on conventional PCR [21,22]. However, to date, no study has performed a genetic characterization of the bacteria.

Therefore, the present study aimed to analyze the genetic diversity of pathogenic leptospires identified in small non-flying mammals in the Atlantic Forest of southeastern Brazil, comparing the identified haplotypes with those described in human and domestic animals to assess the risk of zoonotic transmission and provide new epidemiological inferences.

## 2. Materials and Methods

All experimental procedures were approved by the Ethics Committee on Animal Use of the Oswaldo Cruz Institute (CEUA-IOC, Comissão de Ética no Uso de Animais, Instituto Oswaldo Cruz, protocol number P-70/13-2, license number LW-39/14). Fieldwork was in full compliance with the federal license issued by the Instituto Brasileiro do Meio Ambiente e dos Recursos Naturais Renováveis of the Brazilian Ministry of the Environment (IBAMA/SISBIO, license number 13373-1).

### 2.1. Animal Capture and Study Areas

The capture and taxonomic identification procedures of the small non-flying mammals are shown in [21,22]. Briefly, specimens were captured with Sherman^®^ and Tomahawk^®^ live traps set in sampling transects, 4–10 in each study area, each with 20 traps, evenly spaced 10–20 m apart. The traps were verified and rebaited daily during seven to ten consecutive sampling nights. In total, 265 kidney samples were obtained, one for each host, including the orders Didelphimorphia (*n* = 21) and Rodentia (*n* = 244).

**Figure 1:**
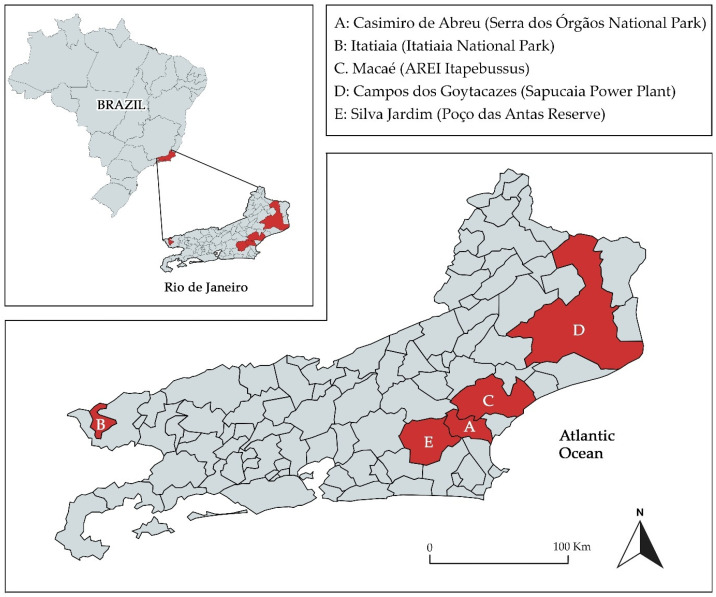
Map of the State of Rio de Janeiro, Brazil, showing the collection sites (municipality/conservation area) of small non-flying mammals (red). The Atlantic Forest is the biome found along the coast of the Atlantic Ocean.

The study area included five different conservation units within the Atlantic Forest in the Rio de Janeiro state, Brazil: (A) Serra dos Órgãos National Park/Casimiro de Abreu; (B) Itatiaia National Park/Itatiaia; (C) Area of Relevant Ecological Interest Itapebussus/Macaé; (D) Sapucaia Power Plant/Campos dos Goytacazes; and (E) Poço das Antas Reserve/Silva Jardim (Figure 1). Areas A, B, and E are strictly protected regions with minimal human interference, while areas C and D are zones with more pronounced anthropogenic activities due to industrial, agricultural, or urban development.

Samples from areas A (*n* = 15), B (*n* = 77) and C (*n* = 34) had been previously subjected to amplification based on the *lipL32* gene. Of these, 63/126 (50%) were positive [21,22]. Samples from areas D (*n* = 52) and E (*n* = 87) were processed in the present study, totaling 139 samples. These were subjected to DNA extraction and molecular diagnosis.

### 2.2. Molecular Diagnosis

All unprocessed small mammal kidney samples (*n* = 139) were subjected to DNA extraction using a DNeasy^®^ Blood & Tissue Kit (Qiagen, Hilden, Germany following the manufacturer’s instructions. For detecting pathogenic *Leptospira* spp., *lipL32* gene-based PCR was performed using primers and conditions described by Hamond et al. (2014) [23]. Each sample set included ultrapure water as a negative control in all reactions, and 10fg of DNA extracted from *Leptospira interrogans* serovar Copenhageni (Fiocruz L1–130) served as a positive control. PCR products were analyzed by 2% agarose gel electrophoresis and visualized under ultraviolet light after staining with GelRed^®^.

### 2.3. Genetic Characterization of Pathogenic Leptospira *spp.*

All samples positive for the *lipL32* gene were subjected to a nested PCR based on the *secY* gene, a genetic marker with good taxonomic resolution, whose use is well established for the genetic characterization of leptospires [24]. Reactions were performed using primers and conditions described in Grillová et al., 2020 [25], with the enzyme Platinum Taq DNA Polymerase kit (Invitrogen, Carlsbad, CA, USA). The amplicons were purified with a PCR Clean-Up System Kit (Promega, Madison, WI, USA) according to the manufacturer’s instructions and intended for sequencing. The reactions were performed in both directions using a Big Dye Terminator v. 3.1 Cycle Sequencing Kit (Applied Biosystems, Foster City, CA, USA) on a 3100 automated DNA sequencer according to the manufacturer’s instructions.

### 2.4. Phylogenetic Analyses

For molecular epidemiology purposes, we used the Single Locus Sequencing Typing (SLST) typing method. For this, sequences of the *secY* gene were selected from GenBank and subjected to phylogenetic analyses with the sequences obtained in the present study. Very short sequences (<400 bp) were excluded to avoid bias. After aligning the sequences in the ClustalX v 2.0 software [26], a maximum likelihood (ML) tree was constructed in MEGA X software v 11.0.13 [27] using the best-fitting DNA substitution model determined by the Bayesian information criterion. Genetic distances were calculated in MEGA X with the model previously determined by the analysis of the data set.

### 2.5. Assessment of Genetic Diversity and Determination of Haplotype Circulation Patterns

The identification of unique haplotypes was performed after screening in the DAMBE software v 7.3.32 [28]. To assess the intraspecific genetic diversity of *Leptospira* spp. populations and evaluate the dynamics of haplotype circulation between different hosts and regions, haplotype networks were constructed from the aligned sequences and scripts in nexus format in the PopART population genetics software using the median-joining inference method [29].

## 3. Results

### 3.1. Molecular Identification of Pathogenic Leptospira *spp.*

Of the 139 samples processed and submitted to molecular diagnosis based on the *lipL32* PCR, 78 (56%) were positive. Those 78 plus the 63 previously positive samples [21,22] were subjected to a nested PCR based on the *secY* genetic marker, totaling 141 DNA samples. It was possible to obtain strong amplicons, suitable for sequencing, from 93 samples.

### 3.2. Genetic Characterization of Pathogenic Leptospira *spp.* Identified

Based on the *secY* gene sequencing, all samples were identified as *L. interrogans* with >99% identity with reference sequences after searching against the BLAST/NCBI platform (accessed 15 January 2025). The majority of hosts were from the order Rodentia (*n* = 83), with few from the order Didelphimorphia (*n* = 10), totaling 10 different genera. From these, *Akodon* and *Mus* had the highest number of positive animals with generated sequences (*n* = 23 and *n* = 21, respectively) (Table 1). The distribution of genetically characterized samples according to location and hosts is shown in Supplementary Table S1.

### 3.3. Phylogenetic Analyses and Haplotype Distribution

**Figure 2:**
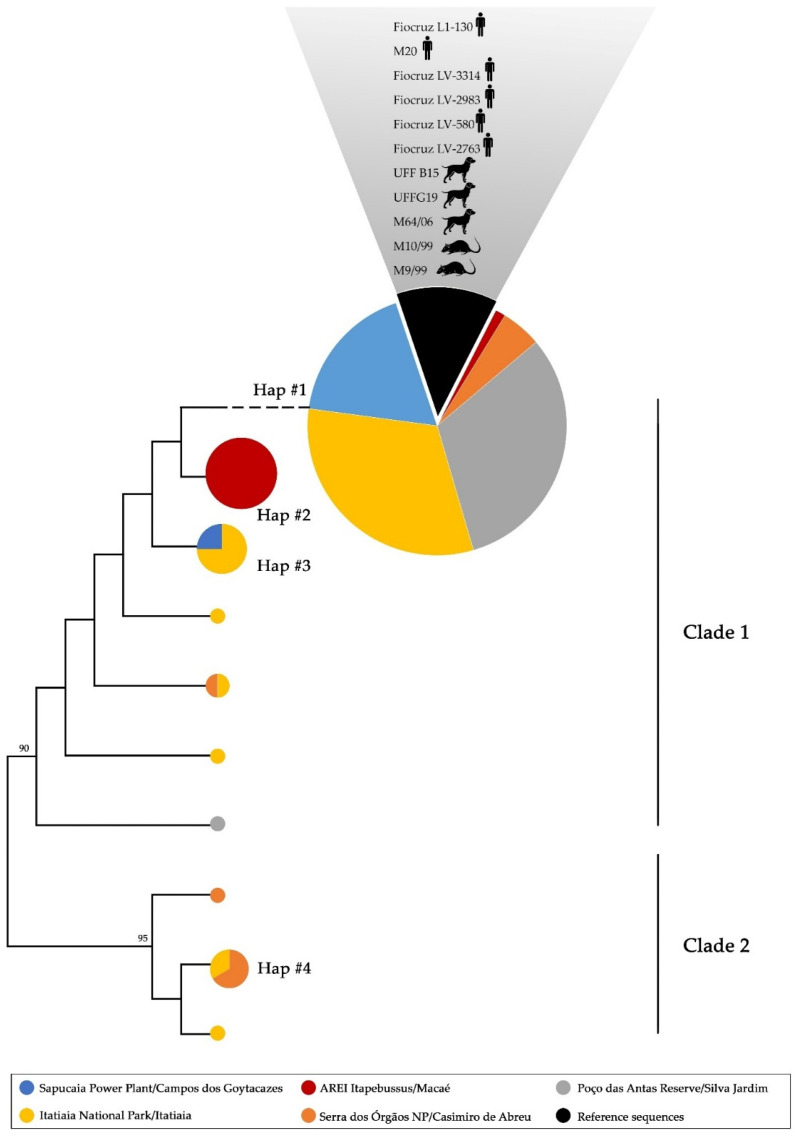
Maximum likelihood (ML) phylogenetic tree and haplogroup distribution inferred from partial *secY* sequences of *L. interrogans*. The area of the circle is proportional to the number of sequences. Colors indicate the place of origin of the sequence (legend). Reference sequences obtained from GenBank were also included, identified in hosts (identified by a vector) and also from the Atlantic Forest (represented in black). AREI: Area of Relevant Ecological Interest; NP: National Park.

The Tamura Nei model (TN92) was the best DNA substitution model determined. Based on this, a high genetic identity was found among the sequences from the present study (TN92 = 0.01 ± 0.01; 99% identity). The phylogenetic analyses and haplotype network demonstrated the presence of four main haplogroups, Hap#1 (*n* = 68), Hap#2 (*n* = 9), Hap#3 (*n* = 5), and Hap#4 (*n* = 4), included in two main clades, with Hap#1, Hap#2, and Hap#3 included in clade 1 and Hap#4 included in clade 2 (Figure 2). This clade included only the sequences from Serra dos Órgãos National Park and Itatiaia National Park, while clade 1 included sequences from all studied sites. Hap#2 was formed exclusively by sequences from AREI Itapebussus. Seven haplotypes were identified as unique, not forming haplogroups (Table 1, Figure 2). There were no specific haplogroups formed according to hosts (Figure 2).

Hap#1 included the vast majority of sequences in the present study, including those from rodents and marsupials from the five regions studied. Furthermore, they presented 100% identity with sequences identified in humans, dogs, and urban rats, all from the Icterohaemorrhagiae serogroup from the same geographical area (Figure 2).

## 4. Discussion

Herein we performed, for the first time, the genetic characterization of pathogenic leptospires identified in 15 species of small non-flying wild mammals from conservation units of the Brazilian Atlantic Forest. A high occurrence was found (56%), which agrees with studies carried out in the same region [21,22]. Another study conducted in small wild rodents from the southeast Brazilian Atlantic Forest, but in rural areas, revealed a lower prevalence (21.3%) and host diversity (five species) based on qPCR diagnosis [30]. This is not surprising, since conservation areas often support greater diversity and abundance of reservoir hosts, greater environmental persistence of the bacteria, low human impact and less use of control measures, greater interactions between species and trophic networks, and less competition with synanthropic species [31]. A study also conducted in conservation areas of the Brazilian Amazon biome revealed a high prevalence of leptospiral DNA in small mammals (44.7%) from 16 different species [32], reinforcing that conservation areas, with their rich biodiversity, can harbor multiple natural reservoirs for *Leptospira*.

Most of the hosts were from the order Rodentia (*n* = 83), with emphasis on the genera *Akodon* (n = 23) and *Mus* (n = 21). The genus *Akodon*, which includes neotropical wild rodents, has been identified as a major carrier of pathogenic *Leptospira* in the Atlantic Forest region of Brazil. Studies have shown that a substantial proportion of *Akodon* spp. are PCR-positive for pathogenic *Leptospira*, highlighting their role as reservoirs in this environment [21,22]. This suggests that *Akodon* rodents contribute to the maintenance and transmission of leptospires in areas where they are prevalent, although their impact may be more pronounced in rural or forested areas rather than urban settings like Rio de Janeiro. Studies on *Akodon* and other sigmodontine rodents in other South American regions, such as the Paraná Delta in Argentina, further support their involvement in the transmission of *Leptospira*. The species *Akodon azarae* was identified as a host for *Leptospira interrogans* serovar Icterohaemorrhagiae, suggesting that these rodents can carry and potentially transmit zoonotic *Leptospira* strains across their geographic range, which includes parts of Brazil [33]. In contrast, the role of *Mus musculus* in transmitting *Leptospira* spp. varies by region. While they can be relevant carriers in certain areas, such as urban settlements in Nairobi, Kenya [34], and rural settings in Puerto Rico [35], their role seems to be less pronounced in urban areas in Brazil, like in Rio de Janeiro, where *Rattus* species are the major reservoirs [36]. Other rodents identified in the present study included *Oligoryzomys nigrips*, *Trinomys marmosa*, *Brucepattersonius* sp., *Delomys altimontanus*, *Delomys dorsalis*, *Oxymycterus* sp., and *Necromys lasiurus*, previously identified as hosts of *Leptospira* in the region [21,22,30].

Regarding the marsupials, *Didelphis aurita*, *Monodelphis* sp., and *Marmosa paraguayana* were herein identified as hosts of *L. interrogans*. Other studies have investigated marsupials, particularly opossums, as hosts of *Leptospira* spp. In a study performed in Southern Brazil, the white-eared opossum (*Didelphis albiventris*) was identified as a reservoir of pathogenic *Leptospira* spp. [37]. Another study examined a broader range of wildlife in Brazil, including opossums, and identified *L. santarosai* as the prevalent species, with a higher risk of infection observed in adult and male opossums when compared to their younger and female counterparts [19]. Additionally, a study conducted in Rio Grande do Sul, Brazil, detected pathogenic *Leptospira* spp. DNA in various wild mammals, including the white-eared opossum. The species *L. borgpetersenii* and *L. interrogans* were identified [38]. These studies collectively highlight the significance of marsupials, particularly opossums, as hosts of *Leptospira* in Brazil, with potential contributions to the maintenance of the bacterial transmission cycle.

Regarding the phylogenetic analyses based on the SLST method applied, it was possible to observe a high genetic identity between the sequences from the present study (TN92 = 0.01 ± 0.01; 99% identity) (Figure 2). This can be explained by the geographic proximity between the studied areas, which present shared environmental features such as similar climatic and ecological conditions (e.g., temperature, humidity, and water bodies). Moreover, some *Leptospira* spp. strains exhibit genetic stability over time, especially if they are well adapted to their host and/or environment [39], which appears to be the case for rodent and marsupial hosts in this region of the Atlantic Forest. Limited selective pressure in certain environments may reduce the rate of genetic divergence [40]. On the other hand, it is important to note that, while in the Atlantic Forest, a low genetic diversity was observed among *Leptospira* spp., a study conducted in the Western Amazon Forest identified three different species, with high intraspecific genetic variation [32]. This greater genetic diversity of *Leptospira* in the Amazon region is probably related to the high biodiversity of hosts and less fragmentation of the habitat. The Atlantic Forest, with its high fragmentation and smaller number of reservoir species, may be subject to a genetic bottleneck effect, limiting the diversity of bacterial lineages [41].

Regarding intraspecific analysis, our results showed the presence of four main haplogroups, not related to hosts or geographic locations, except for Hap#2, which was formed exclusively by sequences from AREI Itapebussus (Figure 2). Importantly, Hap#1 included the largest number of sequences in the present study (*n* = 68) that also comprised *L. interrogans* reference sequences from humans, dogs, and urban rats from the Icterohaemorrhagiae serogroup. It is well known that the clonal subpopulation Fiocruz L1-130 of *L. interrogans* serogroup Icterohaemorrhagiae is widely distributed in Brazil and Latin America in different hosts with a major zoonotic role [42,43]. The data from the present study demonstrate that small wild mammals from the Atlantic Forest are also reservoirs of this strain, which seems to be ubiquitarian in that biome. This outcome raises an important public health concern: *L. interrogans* from the Icterohaemorrhagiae serogroup is the major agent of leptospirosis in humans [8], and rodents in forested areas, particularly in regions like the Atlantic Forest, can act as a direct or indirect link to human infection.

The Atlantic Forest is one of the most biodiverse biomes in the world but is facing severe degradation due to urbanization, agriculture, and logging, with much of its original cover already destroyed [44]. It is the most deforested forest in Brazil, and currently, only 12.4% of the original forest remains [45]. This degradation has a direct impact on local ecology and public health, especially on the transmission of zoonotic diseases such as leptospirosis, which may occur due to different factors: (i) habitat fragmentation and increased human–wildlife interaction; (ii) disruption of ecosystem balance (disturbed trophic interactions and bacterial persistence in altered environments); (iii) increased exposure to contaminated water sources; (iv) changes in biodiversity that may lead to shift in host species; and (v) climate change, which can promote favorable conditions for the spread of the bacteria [46].

## 5. Conclusions

The present study revealed a high genetic identity among pathogenic leptospires circulating in small non-flying mammals from the southeastern Atlantic Forest biome, highlighting the presence of a larger haplogroup that includes a highly virulent strain also detected in humans, dogs, and urban rats (*L. interrogans* serogroup Icterohaemorrhagiae, strain Fiocruz L1-130), and evidenced the zoonotic potential of the identified leptospires. Our results reinforce the role of small mammals, such as rodents and marsupials, as important carriers of *Leptospira interrogans* and draw attention to the Atlantic Forest as an important environment for the circulation and dissemination of the spirochaete. Detecting virulent strains in animals of different species that inhabit forests and peri-urban areas warns of the imminent risk to public health, especially in regions where interactions between humans and wildlife are frequent. This study highlights the urgent need for epidemiological surveillance and control actions in southeast Brazil, aiming to mitigate the transmission of leptospirosis and prevent future outbreaks, especially in highly populated areas from the Atlantic Forest submitted to intense fragmentation and anthropic actions. In this context, a One Health approach is fundamental, integrating human, animal, and environmental health to effectively manage and mitigate the impact of zoonotic diseases such as leptospirosis.

## Figures and Tables

**Table 1:** Haplogroup distribution of *Leptospira interrogans* identified in the present study according to collection site and host.

Collection Site	Host	Haplogroup				
		#1	#2	#3	#4	ND
(A) Serra dos Órgãos NP (*n* = 8)	*Didelphis aurita* (*n* = 1)	1				
	*Akodon montensis* (*n* = 3)	1			1	1
	*Oligoryzomys nigrips* (*n* = 3)	1			1	1
	*Trinomys marmosa* (*n* = 1)	1				
(B) Itatiaia NP (*n* = 34)	*Monodelphis* sp. (*n* = 4)	2		2		
	*Akodon* sp. (*n* = 8)	6			1	1
	*Brucepattersonius* sp. (*n* = 10)	8		1	1	
	*Delomys* sp. (*n* = 6)	3		1		2
	*Oligoryzomys* sp. (*n* = 2)	2				
	*Oxymycterus* sp. (*n* = 4)	3				1
(C) AREI Itapebussus (*n* = 10)	*Marmosa paraguayana* (*n* = 5)	1	4			
	*Akodon cursor* (*n* = 3)		3			
	*Mus musculus* (*n* = 2)		2			
(D) Sapucaia Power Plant * (*n* = 15)	*Mus musculus* (*n* = 15)	14		1		
(E) Poço das Antas Reserve * (*n* = 26)	*Akodon* sp. (*n* = 9)	9				
	*Mus musculus* (*n* = 4)	4				
	*Necromys lasiurus* (*n* = 13)	12				1
Total		68	9	5	4	7

AREI: Area of Relevant Ecological Interest; NP: National Park; ND: not defined. Samples from sites indicated with an asterisk (*) were extracted and amplified based on the *lipL32* gene in the present study. The other samples were only amplified and sequenced for the *secY* gene, as they had already been previously amplified for the *lipL32* gene in previous studies [21,22].

## Data Availability

The data that support the findings of this study are openly available in GenBank under accession numbers PQ999001- PQ999093.

## Supplementary Materials

The following supporting information can be downloaded at https://www.mdpi.com/article/10.3390/tropicalmed10030062/s1, Supplementary Table S1: Complementary information regarding the origin and hosts of pathogenic leptospires from the Brazilian Atlantic Forest genetically characterized in the present study based on the *secY* gene.
