# Supplementary material for: Genetic Characterization and Zoonotic Potential of Leptospira interrogans Identified in Small Non-Flying Mammals from Southeastern Atlantic Forest, Brazil
Source: Trop Med Infect Dis. 2025 Feb 27;10(3):62. doi: 10.3390/tropicalmed10030062 (PMC11945321; doi:10.3390/tropicalmed10030062)
Supplement: Supplementary file 1 [file tropicalmed-10-00062-s001.zip › tropicalmed-3496791-supplementary.pdf]

Collection site	Host	Sample ID	Year of collection	GenBank No.	Haplogroup
A) Serra dos Órgãos NP (*n*=8)	**Didelphimorphia (*n*=1)**				
	Didelphis aurita	19521	2015	PQ999001	#1
	**Rodentia (*n*=7)**				
	Akodon montensis	19490	2015	PQ999002	#1
		19498	2015	PQ999003	-
		19500	2015	PQ999004	#4
	Oligoryzomys nigrips	19480	2015	PQ999005	#1
		19491	2015	PQ999006	-
		19501	2015	PQ999007	#4
	Trinomys marmosa	19639	2015	PQ999008	#1
B) Itatiaia NP (*n*=34)	**Didelphimorphia (*n*=4)**				
	Monodelphis sp.	20013	2015	PQ999009	#1
		20020	2015	PQ999010	#1
		20021	2015	PQ999011	#3
		20035	2015	PQ999012	#3
	**Rodentia (n=30)**				
	Akodon montensis	20044	2015	PQ999013	#1
	Akodon sp.	19974	2015	PQ999014	#1
		19977	2015	PQ999015	#1
		19978	2015	PQ999016	#1
		19983	2015	PQ999017	#1
		19991	2015	PQ999018	#1
		20008	2015	PQ999019	#4
		20016	2015	PQ999020	-
	Brucepattersonius sp.	19973	2015	PQ999021	#3
		19975	2015	PQ999022	#4
		19981	2015	PQ999023	#1
		19997	2015	PQ999024	#1
		20000	2015	PQ999025	#1
		20006	2015	PQ999026	#1
		20019	2015	PQ999027	#1
		20025	2015	PQ999028	#1
		20033	2015	PQ999029	#1
		20036	2015	PQ999030	#1
	Delomys altimontanus	19986	2015	PQ999031	-
	Delomys dorsalis	19982	2015	PQ999032	#3

		19984	2015	PQ999033	-
		19998	2015	PQ999034	#1
		20011	2015	PQ999035	#1
		20047	2015	PQ999036	#1
	Oligoryzomys sp.	20012	2015	PQ999037	#1
		20045	2015	PQ999038	#1
	Oxymycterus sp.	20009	2015	PQ999039	#1
		20017	2015	PQ999040	-
		20023	2015	PQ999041	#1
		20043	2015	PQ999042	#1
C) AREI Itapebussus (*n*=10)	**Didelphimorphia (n=5)**				
	Marmosa paraguayana	18706	2016	PQ999043	#2
		18707	2016	PQ999044	#2
		18709	2016	PQ999045	#1
		18941	2016	PQ999046	#2
		18942	2016	PQ999047	#2
	**Rodentia (*n*=5)**				
	Akodon cursor	18689	2017	PQ999048	#2
		18702	2017	PQ999049	#2
		18713	2017	PQ999050	#2
	Mus musculus	18700	2017	PQ999051	#2
		18705	2017	PQ999052	#2
D) Sapucaia Power Plant (*n*=15)	**Rodentia (*n*=15)**				
	Mus musculus	20111*	2017	PQ999053	#1
		20112*	2017	PQ999054	#1
		20113*	2017	PQ999055	#3
		20114*	2017	PQ999056	#1
		20115*	2017	PQ999057	#1
		20116*	2017	PQ999058	#1
		20117*	2017	PQ999059	#1
		20118*	2017	PQ999060	#1
		20122*	2017	PQ999061	#1
		20123*	2017	PQ999062	#1
		20133*	2017	PQ999063	#1
		20140*	2017	PQ999064	#1
		20142*	2017	PQ999065	#1

		20150*	2017	PQ999066	#1
		20155*	2017	PQ999067	#1
E) Poço das Antas Reserve (*n*=26)	**Rodentia (n=26)**				
	Akodon sp.	19908*	2017	PQ999068	#1
		20205*	2017	PQ999069	#1
		20206*	2017	PQ999070	#1
		20207*	2017	PQ999071	#1
		20208*	2017	PQ999072	#1
		20209*	2017	PQ999073	#1
		20210*	2017	PQ999074	#1
		20211*	2017	PQ999075	#1
		20212*	2017	PQ999076	#1
	Mus musculus	19932*	2017	PQ999077	#1
		19942*	2017	PQ999078	#1
		19946*	2017	PQ999079	#1
		19947*	2017	PQ999080	#1
	Necromys lasiurus	19897*	2017	PQ999081	#1
		20204*	2017	PQ999082	#1
		20213*	2017	PQ999083	#1
		20214*	2017	PQ999084	#1
		20215*	2017	PQ999085	#1
		20216*	2017	PQ999086	#1
		20217*	2017	PQ999087	-
		20218*	2017	PQ999088	#1
		20219*	2017	PQ999089	#1
		20220*	2017	PQ999090	#1
		20231*	2017	PQ999091	#1
		20234*	2017	PQ999092	#1
		20236*	2017	PQ999093	#1

AREI: Area of Relevant Ecological Interest; NP: National Park; Samples indicated with an asterisk (*) were extracted and amplified based on the *lipL32* gene in the present study. The other samples were only amplified and sequenced for the *secY* gene, as they had already been previously amplified for the *lipL32* gene in previous studies (Vieira et al., 2019a; et al., 2019b).
